# Observations and Experiments upon Dr. Jame's Powder: with a method of Preparing, in the Humid Way, a Similar Substance

**Published:** 1801-12

**Authors:** Richard Chenevix


### To Dr. BATTY.

DEAR SIR,

AS the ſubject of the encloſed paper, which has been read at the Royal Society, comes within the province of your Medical and Phyſical Journal, I beg leave to addreſs it to you, in order that this method of preparing, at a cheaper rate than that at which it is uſually ſold, ſo valuable a medicine as James's Powder, may be as public as poſſible. I am, &c.

*Nov.* 12, 1801. RICHARD CHENEVIX.

### *Observations and Experiments upon* Dr. JAMES'S POWDER; *with a Method of preparing, in the humid Way, a similar Substance.* By R. CHENEVIX, Efq. F. R. S. M. R. I. A.

AFTER the obfervations and experiments made by Dr. PEARSON, to inveftigate the nature of Dr. JAMES'S Powder, and prefented by him to this Society, very little remained to be effected or defired, towards a further knowledge of the fubject. But thofe very experiments ferved to fuggeft, that the mode of preparation was far from being the beft that the prefent improved ftate of chemical knowledge might afford; and that, in all probability, a lefs defective compofition might refult from a procefs more conformable to fome improvements, which of late have been advantageoufly applied to pharmaceutic chemiftry.

It may be laid down as a general principle, that, in delicate experiments, whether analytical or fynthetical, fire (that potent and once believed to be univerfal agent) is too precarious in its means, and too uncertain in its application, to be employed with full and conftant fuccefs. And, if it is ftill recurred to, the advantage of promptnefs, and a remnant of ancient cuftom, are the principal reafons. But, where other methods can be devifed to effect the fame combinations, (and the humid way offers many,) every perfon converfant in chemical knowledge will allow the benefit of adopting them. The recent improvement in the mode of preparing calomel, is a ftriking example of fuch falutary corrections being fuccefsfully introduced.

A few obfervations upon the formula according to which Dr. JAMES'S Powder, or the *Pulvis Antimonialis*, is prepared, and upon fome properties of antimony, will place this affertion in a more prominent point of view.

In order to prepare this powder, we are told to take equal weights of bone or hartfhorn fhavings and crude antimony, and calcine them together, at a high temperature: in other words, to take phofphate of lime, which already contains a great excefs of lime, and add to it an oxide of antimony. In this procefs, it has been fuppofed, that the phofphoric acid of the bone or hartfhorn will faturate, not only the lime with which it was originally combined, but, in addition to it, a new portion of metallic oxide, and a new portion of lime. For, what little fulphuric acid might, during the procefs, have been formed by the combuftion of the fulphur of the crude antimony, is diffipated, at a much lower temperature than that to which the powder is expofed.

Every oxide of antimony with which we are acquainted, is volatile at a high degree of heat; it would therefore be hazardous to affert, that it is poffible to preferve always the fame proportion proportion of antimony, whatever care may be employed in directing the operation; and a diffimilarity in the chemical refult muft neceffarily be attended with uncertainty in the medical application.

To this property may be added another, no lefs conducive to error. That portion of oxide of antimony which is not volatilized, becomes, in a great meafure, infoluble in all the acids. What the effect of the gaftric juice may be, upon a fubftance which refifts the action even of nitro-muriatic acid, it is not my purpofe to determine. It is fufficient for me to fay, that, as the quantity of infoluble matter, in a given quantity of Dr. James's Powder, prepared at different times, may vary, the effect of any dofe alfo may differ, according to the proportions of foluble and infoluble matter.

I look upon it as a fortunate circumftance, that thofe experiments and obfervations which I mentioned in the beginning of this Paper, exifted as a ftandard to which I might refer my own attempts, and by which I might eftimate their validity. Dr. Pearson has proved, (as by my own experiments I have found,) that in Dr. James's Powder about 28 per cent. refifted the action of every acid. In examining fome of the *Pulvis Antimonialis* of the London Pharmacopeia, I found the average quantity of infoluble matter to be about 44 per cent. This proportion, however, was liable to confiderable variation.*

The powder here treated of is denominated, by Dr. Pearson, a triple falt, or a real ternary combination of a double bafis, (lime and antimony,) with phofphoric acid. What I have mentioned, with regard to the quantity of acid contained in bone or hartfhorn, as being too fmall to faturate a new portion of thefe bafes, may throw fome doubts upon the poffibility of any fuch combination in the prefent cafe. But I have made fome more direct experiments, which tend to prove, that no fuch combination does exift.

I took fome white oxide of antimony, (formerly called Algaroth Powder,) precipitated by water from muriate of antimony, and heated it for a long time with phofphoric acid. I decanted the liquor, and wafhed the powder that remained. No antimony could be found in the liquor; nor could any traces of phofphoric acid be detected in the refiduary oxide of antimony. I then took a folution of muriate of antimony, and divided it into two equal

* I find, from the information of feveral medical gentlemen, that the *Pulvis Antimonialis* is generally confidered as ftronger than Dr. James's Powder. This feems rather extraordinary, when we confider that the quantity of infoluble matter is greater in the former than in the latter; and would almoft lead us to fufpect it to be the active part of the medicine.

parts: Into one, I poured diftilled water; and, into the other, a folution of phofphate of foda. In each liquor, a copious precipitate was formed; which precipitates, after being well wafhed, were dried. The weight of both was the fame; whereas it is evident that, had any phofphoric acid been combined with the oxide, there would have been an augmentation of weight in that which was precipitated by the folution of phofphate of foda. This precipitate likewife, upon examination, gave no traces of phofphoric acid. From thefe experiments it appears, that there exifts no combination, which can be denominated a phofphate of antimony.

To attempt an explanation of the real nature of the powder here fpoken of, I had recourfe to fome experiments of Mons. Berthollet. By detonating fulphuret of antimony and nitrate of potafh in a crucible, he obtained a mafs, which he reduced to powder, and wafhed. The liquor gave, upon evaporation, a cryftallized falt, which M. Berthollet terms an *antimoniate of potafh*. I never could fucceed in any attempt to form a fimilar combination between the above white oxide of antimony and potafh, owing, I believe, to the fmall quantity of oxygen contained therein, compared with that which is combined with the oxide obtained by detonation. I cannot therefore fay, that the powder in queftion is, in any degree, what M. Berthollet would call an *antimoniate of lime*.

But be the ftate, whether of mixture or of combination, what it may, my purpofe is to endeavour to produce a fubftance, which, from its more certain mode of preparation, may be more equal and conftant in its effects.

Diffolve, together or feparately, in the leaft poffible portion of muriatic acid, equal parts of the forementioned white oxide of antimony and of phofphate of lime.* Pour this folution gradually into diftilled water, previoufly alkalizated by a fufficient quantity of ammonia. A white and abundant precipitate will take place, which, well wafhed and dried, is the fubftitute I propofe for Dr. James's Powder.

---

* In order to procure the phofphate of lime, I diffolved in muriatic acid a quantity of calcined bone, and precipitated by ammonia in its ftate of greateft caufticity. By this means, the excefs of muriatic acid, which held in folution the phofphate of lime, is faturated, and the phofphate is precipitated; but no muriate of lime is decompofed, if the ammonia is quite free from carbonic acid. This is the moft direct method of obtaining phofphate of lime pure. This falt is not decompofed, as fome have afferted, by muriatic acid, but merely diffolved by it. I have been induced to ftate fully thefe particulars, becaufe, from the beneficial effects of this falt in the treatment of rachitis, as propofed by M. Bonhomme, (*Annales de Chimie*, Vol. XVIII. p. 113,) it may become of general ufe. The oxide of antimony I obtained by precipitating, by water, the common butter of antimony of the fhops.

The

The theory of this precipitation is so clear and simple, that it does not require any comment. It may be useful, however, to those who wish to make this preparation, to remark, that it is absolutely necessary that the solution of phosphate of lime and of oxide of antimony, in muriatic acid, should, after being well mixed, be poured *into the alkaline liquor*, in order to obtain a precipitate homogeneous throughout the operation. For, should the alkaline liquor be poured *into the acid solution*, the water of the former would act upon the entire mass of oxide of antimony, while the alkali would precipitate the phosphate of lime only as it saturated the acid which held that salt in solution: thus, the precipitate would contain more antimony in the beginning; and, towards the end, the phosphate of lime would be predominant. For the same reason too, a pure alkali is preferable to its carbonate; for the carbonic acid disengaged, would retain in solution a portion of phosphate of lime.

Whether this composition be a chemical combination or a mixture, I will not take upon me to determine; but, for the reasons above mentioned, in speaking of Dr. James's Powder, I believe it to be merely a very intimate mixture. At all events, it must be more homogeneous than any that can be prepared in the dry way. It is entirely soluble in every acid that can dissolve either phosphate of lime or oxide of antimony separately; and to have it constantly and uniformly the same, no further address in preparing it is required, than to avoid the errors I have mentioned.

As, after some medical trials of the powder, it was suggested to me, that it might be more advantageous to render it somewhat stronger, I prepared another portion, by taking two parts of oxide of antimony and but one of phosphate of lime, and precipitating as above described. The medicinal power was then considerably increased.

Dr. James's Powder is a medicine which has been so long in use, and is so deservedly ranked among the most valuable we possess, that every attempt to render the process for preparing it more simple and more certain, must be allowed to be of some importance. But, whatever reason there was to think, by arguing upon its chemical properties, that I had really succeeded in improving its medicinal virtues, it still remained to be proved, by actual experiment, that the hoped-for success was not merely conjectural. To ascertain this, I gave some of my powder to Dr. Crichton, Dr. Babington, and Mr. Abernethy; gentlemen whose extensive practice and acknowledged skill sufficiently enabled them to judge of its medical properties. They all concur in opinion, that, in its general effects, it agrees with Dr. James's Powder and the *Pulvis Antimonialis*; but, that it is is more mild, and consequently may be given in larger quantities, seldom producing nausea or vomiting, in doses of less than eight or ten grains.

